# Supplementary material for: Does prior knowledge of food fraud affect consumer behavior? Evidence from an incentivized economic experiment
Source: PLoS One. 2019 Dec 3;14(12):e0225113. doi: 10.1371/journal.pone.0225113 (PMC6890166; doi:10.1371/journal.pone.0225113)
Supplement: Attachment — Submitted filename: Report.pdf [file pone.0225113.s002.pdf]

Prior Knowledge of Food Fraud Affect Consumer Behavior?
Evidence from an Incentivized Economic Experiment

PONE-D-19-23258

The authors use an experimental auction to examine how information about olive-oil fraud affects consumers' willingness to pay (WTP) for both high-price and low-price olive oils, while also controlling for prior knowledge of food fraud incidents. Before being treated with information about food fraud, consumers with prior knowledge of fraud are willing to pay less for olive oil than those without prior knowledge of fraud. After being treated with information, both types of consumers reduce their WTP for olive oil, but those with prior knowledge reduce their WTP by less.

The study is well designed and the paper is well written. I have only minor comments.

1) On page 2 you say, "the total number of food fraud incidents in the two years from 2011 to 2012 was 60 percent higher than that in the three decades between 1980 and 2010." That's so stunning, I just want to be clear that I have it right. Does that mean the TOTAL number of incidents reported in that two-year period was 60 percent greater than the TOTAL number of incidents reported in the 30-year period preceding it? Or are you referring to the number of incidents reported per year?

2) I don't understand the long sentence that begins the last paragraph on page 4. In particular, what do you mean when you say, "rather than simply resulting in repeated responses to new information about food fraud incidents"?

3) I don't understand how to interpret the last row in Table 2. Does this mean knowledgeable subjects are only willing to $0.355 for olive oil? If so, which kind of oil – low or high priced? And is this figure referring to WTP before or after being treated with information? Unrelated to that, the third decimal place seems superfluous.

4) In Table 3 please indicate the number of people who came into the experiment with no prior knowledge and with prior knowledge, perhaps be including (N=X) and (N=Y) at the top of each column.

5) I don't understand how you could've run the Hausman test you describe in the last sentence of Section 3. My understanding is that the test statistic is calculated by running the panel regression both ways (i.e., using fixed effects and random effects). But since *Priorknow_fraud* is individual invariant, it would just be absorbed by the individual-specific error terms in a fixed-effects regression. Please clarify.

6) Describing the results of regressions with interaction terms is notoriously tricky. By and large, you do a fine job. However, I think you're wrong when you say, "After receiving information about Italian olive oil fraud, consumers' valuations for Italian EVOO, on average, decreased by $4.42." This is true only for uninformed consumers bidding on low-price oil. Uninformed consumers bidding on high-price oil saw a larger decrease. Namely, $4.42 + $4.65 = $9.07.

Likewise, the change in WTP due to being treated with information were different for informed consumers.

7) Which type of oil do the bars in Figure 1 refer to? High-price oil, low-price oil, or an average of the two?

8) Are the $2.09 and $0.18 differences you report in connection with Figure 1 statistically meaningful? Based on the results from Table 4, the $2.09 difference clearly is. But I'm not sure about $0.18. If I were to guess, I'd say $0.18 isn't statistically meaningfully different from zero. If I'm right, that's a result that supports your argument. Before being treated with information, informed consumers bid significantly less than uninformed consumers. After being treated with information, there's no meaningful difference in WTP.

9) That last paragraph from Section 3 probably belongs in Section 4.

10) You refer to the market for lemons on pages 16 and 17. Does this imply a greater role for third parties (government or otherwise)? Spence's solution to Akerlof's lemon problem is to introduce signals of quality.

11) I didn't realize until I got to the top of page 19 that your "prior knowledge of fraud" variable referred to prior knowledge of ANY kind of food fraud. I'd been assuming you were referring specifically to prior knowledge of fraud in the olive-oil industry. Please be explicit about this earlier in the paper.

12) Throughout the paper, you're using "informed" and "information" to refer to two different things. The first refers to information acquired before the experiment and the second refers to the information people are treated with during the experiment. This is confusing. Consider using a different term in place of "informed." "Knowledgeable" would be one option.
