# Supplementary material for: Does prior knowledge of food fraud affect consumer behavior? Evidence from an incentivized economic experiment
Source: PLoS One. 2019 Dec 3;14(12):e0225113. doi: 10.1371/journal.pone.0225113 (PMC6890166; doi:10.1371/journal.pone.0225113)
Supplement: Attachment — Submitted filename: Response to Reviewers.docx [file pone.0225113.s003.docx]

Prior Knowledge of Food Fraud Affect Consumer Behavior?

Evidence from an Incentivized Economic Experiment

PONE-D-19-23258

**Reviewer# 1** The authors use an experimental auction to examine how information about olive-oil fraud

affects consumers’ willingness to pay (WTP) for both high-price and low-price olive oils, while

also controlling for prior knowledge of food fraud incidents. Before being treated with

information about food fraud, consumers with prior knowledge of fraud are willing to pay less

for olive oil than those without prior knowledge of fraud. After being treated with information,

both types of consumers reduce their WTP for olive oil, but those with prior knowledge reduce

their WTP by less.

The study is well designed and the paper is well written. I have only minor comments.

1) On page 2 you say, “the total number of food fraud incidents in the two years from 2011 to

2012 was 60 percent higher than that in the three decades between 1980 and 2010.” That’s so

stunning, I just want to be clear that I have it right. Does that mean the TOTAL number of

incidents reported in that two-year period was 60 percent greater than the TOTAL number of

incidents reported in the 30-year period preceding it? Or are you referring to the number of

incidents reported per year?

Response to reviewer: Thank you for pointing this out. There was an English-language error. It should have been “60 percent as high” in 2011-12 as in the three preceding decades. We have corrected the text.

2) I don’t understand the long sentence that begins the last paragraph on page 4. In particular,

what do you mean when you say, “rather than simply resulting in repeated responses to new

information about food fraud incidents”?

Response to reviewer: We aimed to draw a distinction between long-term changes in consumer valuation that would show up even in the absence of information—which we elicit in the baseline round—versus identifying “acute” responses to information about food fraud. We have removed the text “rather than simply resulting in repeated responses to new information about food fraud incidents;” we make this point elsewhere more clearly.

3) I don’t understand how to interpret the last row in Table 2. Does this mean knowledgeable

subjects are only willing to $0.355 for olive oil? If so, which kind of oil – low or high priced? And is this figure referring to WTP before or after being treated with information? Unrelated to that, the third decimal place seems superfluous.

Response to reviewer: This number (0.355) represents the proportion of participants who reported having prior knowledge of food fraud. We see that it is potentially confusing to have one non-WTP-related variable reported in a table otherwise dedicated to WTP values, so we have removed this line from the table. We have added a new first sentence to section 3, which reads “Slightly more than one-third (36 percent) of participants reported having prior knowledge of food fraud, while 64 percent of participants reported no knowledge of food fraud.” We have also removed the third decimal place from all values reported in table 2 based on the reviewer’s recommendation.

4) In Table 3 please indicate the number of people who came into the experiment with no prior

knowledge and with prior knowledge, perhaps be including (N=X) and (N=Y) at the top of each

column.

Response to reviewer: We thank the reviewer for this suggestion. We have included this information in the table. The number of participants with no prior knowledge was 69, while the number with prior knowledge was 38.

5) I don’t understand how you could’ve run the Hausman test you describe in the last sentence

of Section 3. My understanding is that the test statistic is calculated by running the panel

regression both ways (i.e., using fixed effects and random effects). But since Priorknow_fraud is

individual invariant, it would just be absorbed by the individual-specific error terms in a fixed effects regression. Please clarify.

Response to reviewer: The reviewer is correct. The Hausman test was conducted in the context of analyzing the willingness to pay data before/after participants were exposed to information and the language was accidentally pulled from a dissertation. We have deleted the text.

6) Describing the results of regressions with interaction terms is notoriously tricky. By and large,

you do a fine job. However, I think you’re wrong when you say, “After receiving information

about Italian olive oil fraud, consumers’ valuations for Italian EVOO, on average, decreased by

$4.42.” This is true only for uninformed consumers bidding on low-price oil. Uninformed

consumers bidding on high-price oil saw a larger decrease. Namely, $4.42 + $4.65 = $9.07. Likewise, the change in WTP due to being treated with information were different for informed

consumers.

Response to reviewer: We thank the reviewer for catching this. We have added text clarifying that this value refers to uninformed consumers’ bids for the low-price-range EVOO and add text noting that their valuation decreased more for high-price-range EVOO. The text now reads, “After receiving information about Italian olive oil fraud, uninformed consumers’ valuations for low-priced Italian EVOO decreased by $4.42, which is statistically significant at the 1% level, while their WTP for high-priced Italian EVOO fell by $9.07.”

7) Which type of oil do the bars in Figure 1 refer to? High-price oil, low-price oil, or an average

of the two?

Response to reviewer: Figure 1 presents the average willingness to pay across low and high-priced EVOOs. We have added text in the figure title to make this clear. The figure now reads, “Estimated WTP for a participant with average characteristics with and without prior knowledge of food fraud before and after receiving information about mislabeling scandals in the Italian EVOO industry, averaged across low-price and high-price-range EVOOs.”

8) Are the $2.09 and $0.18 differences you report in connection with Figure 1 statistically

meaningful? Based on the results from Table 4, the $2.09 difference clearly is. But I’m not sure

about $0.18. If I were to guess, I’d say $0.18 isn’t statistically meaningfully different from zero.

If I’m right, that’s a result that supports your argument. Before being treated with information,

informed consumers bid significantly less than uninformed consumers. After being treated with

information, there’s no meaningful difference in WTP.

Response to reviewer: We thank the reviewer for this suggestion. We estimated the mean WTP of knowledgeable and unknowledgeable participants with and without information about Italian olive oil fraud. Results show that before being exposed to the information about EVOO mislabeling, knowledgeable consumers bid less than unknowledgeable consumers, which is statistically significant at the 5% level. However, after being treated with information, there is no statistically significant difference in WTP of knowledgeable and unknowledgeable participants. We have added bars to figure 1 that represent 95% confidence intervals and added discussion to page 15, which reads, “As depicted in figure 1, before receiving any information about Italian olive oil fraud, participants who reported prior knowledge of food fraud were willing to pay, on average, $1.60 less for Italian EVOO than participants without prior knowledge of food fraud. This result is statistically significant at the 5% level. However, after receiving information about Italian olive oil fraud, there is no statistically significant difference in WTP of participants with and without prior knowledge of food fraud. In other words, before being treated with Italian olive oil fraud information, knowledgeable consumers bid significantly less than unknowledgeable consumers. After being treated with Italian olive oil fraud information, there is no meaningful difference in WTP. Therefore, figure 1 further reinforces our key results that prior exposure to information about food fraud is an important factor in explaining consumer behavior in the presence of food fraud.”

9) That last paragraph from Section 3 probably belongs in Section 4.

Response to reviewer: Thank you for the suggestion. We have moved the text to the discussion.

10) You refer to the market for lemons on pages 16 and 17. Does this imply a greater role for

third parties (government or otherwise)? Spence’s solution to Akerlof’s lemon problem is to

introduce signals of quality.

Response to reviewer: We thank the reviewer for the opportunity to expand on this point. We have added text to the paragraph in which we mentioned the market for lemons. The added text reads, “While a solution to the asymmetric information problem already nominally exists in the market for EVOO—that is, quality certification, food producers are able to fraudulently label their products due to a lack of monitoring and oversight by the organizations responsible for certification systems, which may be governmental or private entities. Increasing the amount of funding available to monitor for mislabeled or adulterated products may be necessary to reduce the number of incidents of food fraud and maintain consumer trust in food labeling systems.”

11) I didn’t realize until I got to the top of page 19 that your “prior knowledge of fraud” variable

referred to prior knowledge of ANY kind of food fraud. I’d been assuming you were referring

specifically to prior knowledge of fraud in the olive-oil industry. Please be explicit about this

earlier in the paper.

Response to reviewer: We thank the reviewer for pointing this out. We have added text at the first mention of prior knowledge to clarify (on page 3). That sentence now reads, “After participants submitted their valuation in the first round, they completed a short survey that included questions about prior knowledge of any—not solely EVOO-related—food fraud incidents.”

12) Throughout the paper, you’re using “informed” and “information” to refer to two different

things. The first refers to information acquired before the experiment and the second refers to

the information people are treated with during the experiment. This is confusing. Consider

using a different term in place of “informed.” “Knowledgeable” would be one option.

Response to reviewer: We thank the reviewer for the helpful suggestion. We have changed *informed* to *knowledgeable* and *uninformed* to *unknowledgeable* throughout the manuscript.

**Reviewer #2:** This article examines the impact of prior knowledge about food fraud (in labeling) on consumer willingness to pay for Olive Oil. The authors find that consumers with prior knowledge of food fraud have a lower WTP as a baseline, and therefore respond less to an information treatment about food fraud. The paper is overall well written and the experimental methods are sound. The manuscript could just use some tightening and clarity.

1) The paper starts in a place that assumes a reader knows about, and cares about, food fraud. The introduction may be an opportunity to educate readers about what food fraud is – I recommend including some media-worthy recent examples. The evidence provided on the magnitude of the problem (i.e. number of large food frauds per year) could be moved up to the first paragraph (I was looking for that information early on). The severity of the problem for consumers could also be addressed – have cases of food fraud led to illness?

Response to reviewer: We thank the reviewer for this suggestion and have added an initial paragraph that discusses the health and economic consequences of food fraud. We also moved the text about the number of food fraud incidents up to the second paragraph.

2) Did you ask specifically about food fraud in the EVOO market? If so, are there differences in ‘informed consumers’ vs. ‘informed about EVOO issues consumers’? From Table 2 it appears you asked familiarity as a (y/n) question, correct? Why not a Likert or other scale? Wording of the actual question familiarity question would be helpful.

Response to reviewer: We asked about knowledge of any type of food fraud rather than specifically about food fraud in the olive oil market. In writing this question, we were weighing trading off specificity of prior awareness of food fraud with sample size considerations—the more specific the question about food fraud, the smaller the sample of participants who would have been aware of the fraud. In light of these considerations, we asked the more expansive question because we did not want to exclude the possibility that consumers exposed to food fraud in general might lose trust broadly in the food system and change their behavior. This likely represents a conservative estimate of the variable since someone aware of food fraud in a different sector may have valued olive oil more highly than someone aware of EVOO fraud. Concerns about the length of the survey led us not to ask both versions of the question.

The wording of the actual question was: “Had you heard about food fraud before coming to the study today?” We have added the text of this question to the manuscript on page 6.

3) Computer interface? (note on what you used)

Response to reviewer: We have added a note about the computer interface. The experiment was programmed using Qualtrics. This has been added on page 6.

4) On page 11, you could improve the link between Table 3 and the text. It feels repetitive to restate your control variables after the table, and yet because the variable names are new (and look like STATA code names), I felt that I needed to look again at the table.

Response to reviewer: We thank the reviewer for this note. We have attempted to standardize variable names in the tables and text.

5) Given the difference in age across the knowledge and no knowledge groups – please consider whether an interaction variable may be appropriate.

Response to reviewer: We thank the reviewer for the suggestion. We had not added an interaction term because age is not significant even as an un-interacted variable, providing evidence that it does not play an important role in explaining EVOO valuation.

6) I think you missed the opportunity for some additional analysis that may be of interest: You indicated you had information about where they purchased olive oil. To the industry, I think it would be interesting to see if place of purchase was related to prior fraud knowledge and/or engagement with the information treatment. Do people seek out specialty stores? Do people who buy from specialty stores have a higher WTP because they assume those stores have done their homework on the products they sell, and therefore are less moved by any additional information?

Response to reviewer: We thank reviewer for this suggestion. We did collect information about where participants generally purchase olive oil. We ran the model including this variable and found no statistically significant relationship between participants’ WTP and olive oil purchasing location. Moreover, there is no statistically significant relationship between prior knowledge of food fraud and olive oil purchasing destination. We have, therefore, excluded it from the analysis. We also removed the reference to the variable from the text.

7) Is the WTP of prior knowledge group and the WTP of the no-knowledge group after information label statistically different or the same? You have reported the WTPs but I remain unclear whether these are statistically different. [Figure 1, error bars may answer my question?]

Response to reviewer: We thank the reviewer for the helpful suggestion. The other reviewer had a similar question. In response, we have added bars to figure 1 that represent 95% confidence intervals and added discussion to page 15, which reads, “As depicted in figure 1, before receiving any information about Italian olive oil fraud, participants who reported prior knowledge of food fraud were willing to pay, on average, $1.60 less for Italian EVOO than participants without prior knowledge of food fraud. This result is statistically significant at the 5% level. However, after receiving information about Italian olive oil fraud, there is no statistically significant difference in WTP of participants with and without prior knowledge of food fraud. In other words, before being treated with Italian olive oil fraud information, knowledgeable consumers bid significantly less than unknowledgeable consumers. After being treated with Italian olive oil fraud information, there is no meaningful difference in WTP. Therefore, figure 1 further reinforces our key results that prior exposure to information about food fraud is an important factor in explaining consumer behavior in the presence of food fraud.”
